# Prevalence of Bluetongue and the distribution of *Culicoides* species in northern and southern regions of Kazakhstan in 2023–2024

**DOI:** 10.3389/fvets.2025.1559636

**Published:** 2025-03-06

**Authors:** Kuandyk Zhugunissov, Dias Muzarap, Nuraiym Sarsenkulova, Muratbay Mambetaliyev, Sanat Kilibayev, Moldir Azanbekova, Marzhan Kenzhebayeva, Shalkar Tabys, Madina Abayeva, Aibarys Melisbek, Nurkuisa Rametov, Kulyaisan Sultankulova, Shawn Babiuk, Aruna Ambagala, Aslan Kerimbayev

**Affiliations:** ^1^Research Institute for Biological Safety Problems, Gvardeisky, Kazakhstan; ^2^Institute of Ionosphere, Almaty, Kazakhstan; ^3^Kazakh National Research Technical University named after K.I. Satbayev, Almaty, Kazakhstan; ^4^National Centre for Foreign Animal Disease, Canadian Food Inspection Agency, Winnipeg, MB, Canada; ^5^Department of Immunology, University of Manitoba, Winnipeg, MB, Canada

**Keywords:** bluetongue, Kazakhstan, *Culicoides*, serology, RNA detection

## Abstract

**Introduction:**

Bluetongue virus (BTV) is a significant vector-borne pathogen affecting ruminants, leading to substantial economic losses, and adversely impacting livestock production worldwide. Recently, Bluetongue (BT) has emerged as a growing concern for European and Asian countries, including Kazakhstan. This study examines the prevalence and distribution of BTV in Kazakhstan during 2023-2024, providing up-to-date information on its occurrence in livestock and *Culicoides* species. The findings aim to contribute to better understanding and management of BT in the region.

**Methods:**

A total of 972 whole blood and 972 serum samples were collected from cattle, sheep, and goats in the southern and northern regions of Kazakhstan, alongside 11,859 *Culicoides* midges in the autumn of 2023 and Spring of 2024. The serum samples were tested for BT virus (BTV)-specific antibodies using ELISA, while the whole blood and *Culicoides* specimens were analyzed for BTV RNA by Real-time RT-PCR (rRT-PCR). Morphological and molecular identification of *Culicoides* species was also conducted.

**Result:**

The overall seroprevalence of BTV in Southern Kazakhstan increased across all animal species in 2024 compared to 2023, with goats showing the most notable rise (from 3.8% to 29.5%). In the northern regions, seroprevalence remained zero in 2023 but reached 10.0% in cattle by 2024. rRT-PCR results confirmed active virus circulation, with rRT-PCR-positive samples significantly higher in 2024, especially among goats (from 4.2% in 2023 to 62.0% in 2024) and cattle (from 9.2% to 34.4%). Based on morphology, nine species of *Culicoides* midges were identified, including *C. obsoletus* a known BTV vector in European countries. Four of them were genetically confirmed, and BTV RNA was detected in all four species (*C. miutissimus, C. sphagnumensis, C. newsteadi*, and *C. pectipennis*), suggesting their potential vectorial role in BTV transmission.

**Discussion:**

This study provides new insights into the epidemiology of BT in Kazakhstan and serves as a valuable resource for veterinary professionals. The findings emphasize the need for continued surveillance and vector control strategies to mitigate the spread of BTV in the region.

## Introduction

*Culicoides* biting midges are the smallest blood-sucking two-winged insects (1–4 mm) in the order Diptera. They belong to the family Ceratopogonidae and have mottled wings with dark patterns that can be used to identify the species (1).

*Culicoides* are important vectors for arboviruses in both animals and humans. The *Culicoides* midges transmit bluetongue (BT), an infectious, non-contagious, vector-borne hemorrhagic viral disease of wild and domestic ruminants. BT is characterized by fever, inflammatory-necrotic lesions in the oral cavity, tongue, the digestive tract, and the epithelium of the coronary band, along with degenerative changes in skeletal and cardiac muscles, subintimal hemorrhage in the pulmonary artery, oedema of the lungs and pericardial, pleural, and abdominal effusions (2, 3). The causative agent, BT virus (BTV) is a double-stranded RNA virus that belongs to the genus *Orbivirus* in the family *Reoviridae*. The genome of BTV is composed of 10 segments of dsRNA. This segmented structure allows the virus to undergo reassortment, leading to the emergence of variants with novel biological characteristics (4). Currently, 29 serotypes of BTV are known to exist (5).

The distribution of BTV has traditionally been observed within the geographical belt between latitudes 40° N and 35° S (6), corresponding to the habitat of *Culicoides* vectors (7). The mild, warm climates of Africa, southern Europe, and Southeast Asia favor the year-round activity of *Culicoides*, facilitating the overwintering of the BTV. Over the past 20 years, the distribution of BTV in Europe has changed significantly. Global climate change has influenced the emergence of different serotypes in geographic areas above 50° and identification of new *Culicoides* vector species (8–10). Currently, the disease has been reported on all continents, with a noticeable trend of expanding to more northern regions (8, 9, 11).

The unprecedented spread of BTV in Europe (6 serotypes across 12 EU countries since 1998), is believed to be driven by the global warming. Reports of BTV detection in countries neighboring Kazakhstan (12–14), has prompted a renewed focus on the epidemiology of BT, and the *Culicoides* vectors in the region.

Historically Kazakhstan was designated as a BT free country. However, serological studies using samples collected during 1996–1998 showed 23.2% BT seroprevalence in livestock (15, 16). Recently Zhigailov et al., reported the detection of BTV antibodies in livestock and BTV genetic material in sheep and *Culicoides* midges in the southeastern region of Kazakhstan. Through molecular genetic analysis and partial sequencing, they determined that the BTV belonged to the “western” topotype of the BTV-9 strain (17). In another study, using mathematical modeling showed that the transmission of BTV in Kazakhstan is not possible between October and March. The winter in Kazakhstan prevents the transmission of BT during this time. Assuming there are endemic BTV competent *Culicoides* midges in Kazakhstan, the modeling predicted risk of BT appearing in the south of Kazakhstan in April and spread north reaching maximum levels in northern Kazakhstan in July, decline in September and disappear by October (18).

Currently, systematic studies of the range and density of the primary vectors of BTV infection in Kazakhstan are either not conducted or are limited to serological monitoring studies. In this study we report, based on the samples collected during 2023–2024, seroprevalence of BT in livestock, the *Culicoides* species associated and their potential role as BTV vectors in Northern and Southern region of Kazakhstan.

## Materials and methods

### Collection and transport of animal biological samples

**Figure 1:**
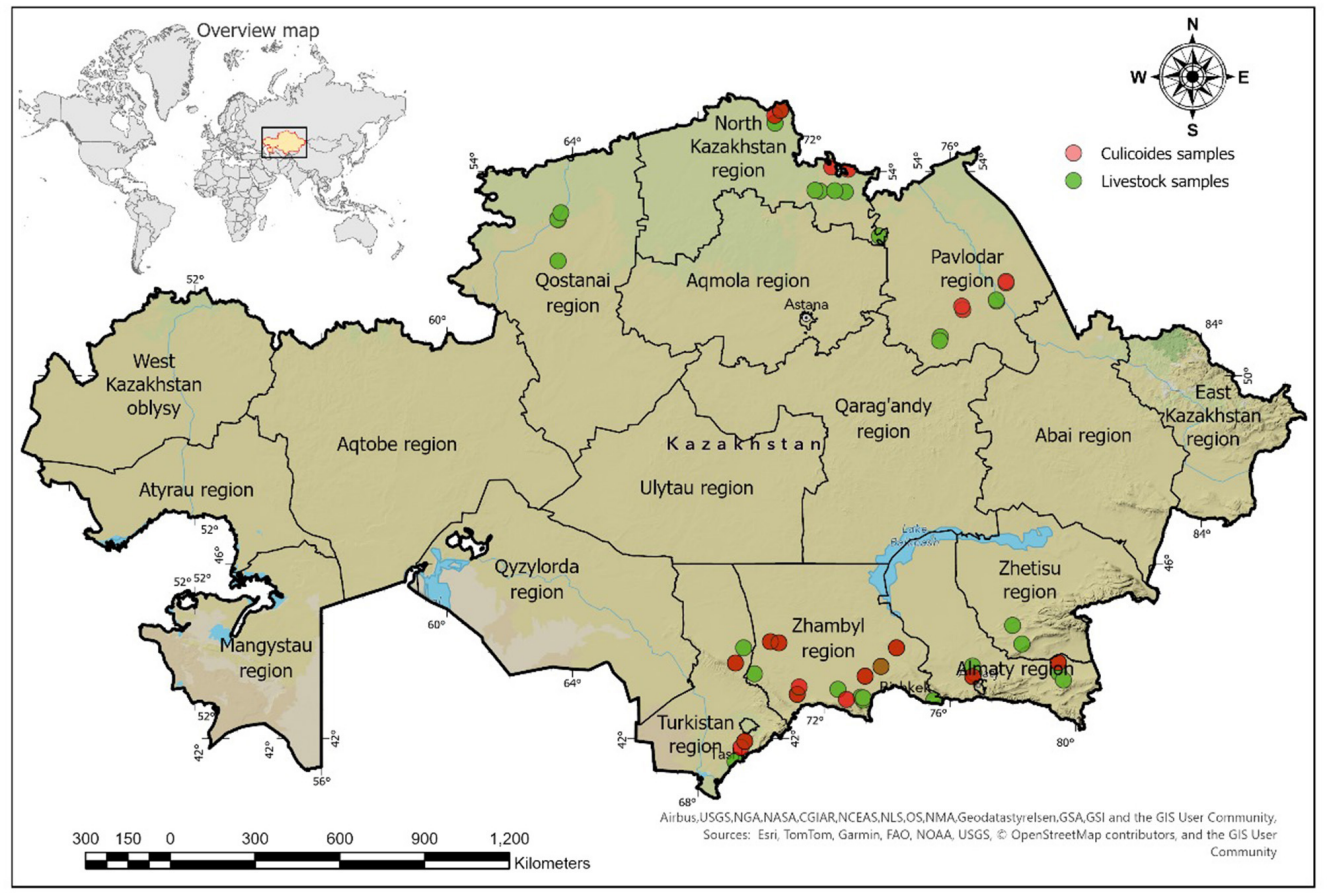
Map of Kazakhstan indicating sampling locations for livestock and *Culicoides* spp.

A total of 972 whole blood and serum samples were randomly collected from clinically healthy sheep, goats, and cattle from the southern regions of Kazakhstan, namely Zhetysu, Almaty, Zhambyl, and Turkestan and the northern regions of Kazakhstan, namely Kostanay, Pavlodar, and North Kazakhstan during August–September 2023 and May–June 2024 (Figure 1). The sample sizes and volumes were determined according to OIE guidelines (19), with modifications based on prevalence levels (Supplementary Table 1) (20).

### Mapping sampling sites using ArcGIS pro

The GPS coordinates of sampling locations were imported and mapped using the WGS 84 projection (EPSG: 4326), and ArcGIS Pro (21) was used to compile and generate the maps. Briefly, a layer of sampling points was created and overlaid on satellite imagery (22). The results were exported as a map with a legend and geographic grid for inclusion in the publication.

### *Culicoides* collection method

*Culicoides* were captured utilizing specialized CDC light traps containing ultraviolet lamp (Light trap, LI-MR-512, BioQuip, USA). The trapping was conducted in the evening at sunset, under calm and windless conditions, in wet, marshy regions adjacent to pastures and livestock facilities. The collected specimens were then transferred to 70% ethanol solution and stored at −20°C for subsequent sorting and morphological identification.

### ELISA

BTV antibodies were detected using the ID Screen^®^ BT Competition kit (Ref: BTC-5P, ID-Vet, France), a competitive ELISA with 96.5% sensitivity and 99.3% specificity, designed to identify anti-VP7 antibodies in serum or plasma from cattle, sheep, and goats (23). Testing and result interpretation followed the manufacturer's instructions.

Optical density (OD) was measured at 450 nm using a Chromate^®^-4300 reader (Awareness Technology, USA). Samples were classified as positive if their OD was <40% of the mean negative control value (S/N) and negative if OD was ≥40% of the mean negative control value (S/N).

### RNA extraction

Viral RNA was extracted from whole blood using Qiagen RNeasy Mini Kit (Cat number, Qiagen, Hilden, Germany), in accordance with the manufacturer's guidelines. Briefly, 560 μl of AVL buffer and 5.6 μl of carrier RNA were added to 140 μl of the whole blood sample, followed by thorough mixing at room temperature for 10 min. Next, 560 μl of 96% ethanol was added, and mixed thoroughly. The resulting solution was then transferred to RNA binding columns and centrifuged at 8,000 rpm for 30 s. The columns were then washed with 700 μl of AW1 buffer each, followed by 500 μl of AW2 buffer involving by centrifugation at 8,000 rpm for 30 s. To remove any residual buffer, the columns were centrifuged at 12,000 rpm for additional 1 min, and viral RNA was eluted by adding 40 μl of AVE buffer, followed by centrifugation at 8,000 rpm for 1 min. The purity and concentration of the isolated RNA were assessed spectrophotometrically at 260/280 nm, with samples yielding an A260/A280 ratio between 1.8 and 2.0 considered pure and suitable for subsequent rRT-PCR use.

### rRT-PCR setup

ID Gene™ Bluetongue Duplex rRT-PCR (Cat number: IDBTV-50, Innovative Diagnostics, Grabels, France) was conducted using a Rotor-Gene Q thermal cycler (Qiagen), and the results were analyzed using Rotor-Gene Q software version 1.8.187.5. rRT-PCR setup was performed in accordance with the ID Gene BT Duplex kit instructions and the following thermal cycling conditions were followed: reverse transcription at 45°C for 10 min, initial denaturation at 95°C for 10 min followed by 40 amplification cycles (denaturation at 95°C for 15 s, annealing and extension at 60°C for 30 s). Detection of the fluorescent signal occurred during the annealing phase in the “Green” channel. Upon completion of the reaction, the amplification curve was analyzed, and a final report was generated. The threshold line was established at a maximum level of 0.05. The reliability of the obtained data was verified through appropriate values of negative and positive controls.

### Morphological and genetic identification of midges

Morphological identification of midges was performed based on wing patterns and size (24), under a stereomicroscope (Optika^®^, Model SZO-T, S/N 651480-482, Ponteranica, Italy). Identification keys (25, 26) and the interactive specialized software *Culicoides* Xper2 Version 2.0 (www.iikculicoides.net) were utilized for precise species determination.

For molecular characterization of the *Culicoides* species, DNA from individual midge specimen was extracted using the DNeasy Blood & Tissue Kit (Qiagen, Ref: 69506, Hilden, Germany). Briefly, each midge specimen was added to ATL buffer, supplemented with Proteinase K, and mixed with 200 μl of Buffer AL. The mixture was vortexed for 15 s and incubated at 56°C for 10 min to ensure complete lysis. Subsequently, 200 μl of 96%−100% ethanol was added, and the mixture was vortexed for another 15 s. The lysate was transferred to a DNeasy Mini Spin Column and centrifuged at 6,000 × g (8,000 rpm) for 1 min. The column was then washed with 500 μl of Buffer AW1, centrifuged at 6,000 × g for 1 min, followed by a second wash with 500 μl of Buffer AW2, and centrifuged at 20,000 × g (14,000 rpm) for 3 min. DNA was eluted with 200 μl of nuclease-free water, incubated for 1 min, and centrifuged at 6,000 × g for 1 min. The eluted DNA was stored at −20°C until further analysis. The 28S rDNA is a molecular marker that can be used to differentiate closely related *Culicoides* species. The primers (Forward 28S rDNA 28S_C'1 5′-ACCCGCTGAATTTAAGCAT-3′, and Reverse 28S_D2 5′-TCCGTGTTTCAAGACGGG-3′) were used to amplify 28S rRNA gene from each specimen (27–29). The cycling conditions used were; initial denaturation at 98°C for 30 s, succeeded by 35 cycles comprising denaturation at 98°C for 10 s, annealing at 55–58°C for 90 s, and extension at 72°C for 45 s. This process concluded with a final extension at 72°C for 5 min. The PCR products, ~700 bp in length, were subsequently assessed using gel electrophoresis on a 1.5% agarose gel and subjected to Sanger sequencing.

### Sanger sequencing

Sanger sequencing method, which employs terminating dideoxynucleotides on a 3,500 × l Genetic Analyzer (Applied Biosystems, Thermo Fisher Scientific, Japan. Serial number: 35395-010) was used to sequence the 28S rDNA amplicons. This process utilizes the BigDye™ Terminator v3.1 Cycle Sequencing Kit (Thermo Fisher Scientific), following the manufacturer's protocols. Capillary electrophoresis is facilitated using POP-7 (Thermo Fisher Scientific) as the polymer. DNA termination products are produced through cyclic sequencing. To purify the DNA from unbound dyes, gel filtration is carried out using Centri-Sep columns or with CleanSeq Reagent, in accordance with the kit instructions. The sequencing operations on the sequencer conform to the guidelines provided with the instrument. The resulting chromatograms were analyzed using the Sequencher software package (Gene Codes Corporation, Ann Arbor, MI), and the sequences were analyzed against the GenBank (NCBI) database with MEGA11 software to identify midge species based on percent identity (30).

### Statistical analysis

The statistical analysis was performed using GraphPad Prism version 9 (GraphPad Software, Inc., La Jolla, CA, U.S.A.). In a univariable analysis, the chi-square test was performed to examine the relationships between risk factors and BTV seroprevalence. The chi-square test was used to assess the strength of association between independent variables, effectively evaluating differences in BTV prevalence across different regions and animal species in our study.

Using multivariable logistic regression, potential risk factors with *p*-values < 0.05 were further evaluated. Logistic regression analysis was employed to assess the relationship between the dependent variable (presence of BTV infection) and independent variables (e.g., age, sex, geographic location). This method allowed for the simultaneous consideration of multiple variables. For the outcome variable, a multivariable model was constructed using logistic regression analysis. Confidence intervals (95% CI) and odds ratios (OR) were calculated.

## Results

### Prevalence of BTV in animals

Thus far, animals showing BT clinical signs have never been reported in the northern regions. However, in the southern regions, sheep with clinical manifestations of BT have been reported. In this study, all the samples (a total of 972 samples of whole blood and sera) were collected from clinically healthy animals in the southern and northern Kazakhstan in the autumn of 2023 and Spring of 2024. This included 642 samples from sheep, 87 from goats, and 243 from cattle. All sera samples were evaluated by ELISA for BTV-specific antibodies (Table 1). The overall serology results revealed a higher number of positive samples in the southern regions compared to the northern regions (*p* < 0.0001) (Supplementary Table 2). When the data was analyzed based on animal species, the overall seroprevalence was 4.2% (95% CI: 2.9–6.0) among sheep, 21.8% (95% CI: 14.4–31.6) among goats, and 14.4% (95% CI: 10.5–19.4) among cattle (Supplementary Table 3). When compared the year of collection, seroprevalence in the southern regions for 2023 Autumn was 5.4% (95% CI: 2.9–9.9) among sheep, 4.2% (95% CI: 0.7–20.2) among goats, and 10.5% (95% CI: 5.4–19.4) among cattle. In 2024 Spring, the seroprevalence was 6.8% (95% CI: 4.3–10.5) for sheep, 36.0% (95% CI: 24.1–49.9) for goats, and 22.9% (95% CI: 15.6–32.3) for cattle (Supplementary Table 4).

**Table 1:** Detection of seropositive and rRT-PCR-positive animal species in the southern and northern regions of Kazakhstan for the years 2023–2024.

**Animal species**	**Year and season**	**Region**	**Oblast**	**Animals tested**	**Seropositive samples (*n*, %)**	**95% Confidence intervals (CI)**	**rRT-PCR-Positive samples (*n*, %)**	**95% Confidence intervals (CI)**
Sheep	2023, Autumn	Southern regions	Zhetysu	40	1 (2.5)	0.4–12.8	5 (12.5)	5.5–26.1
			Almaty	30	0 (0.0)	0.0–11.3	0 (0.0)	0.0–11.3
			Zhambyl	50	3 (6.0)	2.1–16.2	2 (4.0)	1.1–13.5
			Turkestan	47	5 (10.6)	4.6–22.6	5 (10.6)	4.6–22.6
	2024, Spring		Zhetysu	60	0 (0.0)	0.0–6.0	6 (10.0)	4.7–20.1
			Almaty	85	0 (0.0)	0.0–4.3	12 (14.1)	8.3–23.1
			Zhambyl	78	10 (12.8)	7.1–22.0	15 (19.2)	12.0–29.3
			Turkestan	42	8 (19.0)	9.9–33.3	12 (28.6)	17.1–43.6
	2023, Autumn	Northern regions	Qostanay	39	0 (0.0)	0.0–8.9	0 (0.0)	0.0–8.9
	2024, Spring		Pavlodar	89	1 (1.1)	0.2–6.1	0 (0.0)	0.0–4.1
			North Kazakhstan	82	0 (0.0)	0.0–4.5	0 (0.0)	0.0–4.5
Goat	2023, Autumn	Southern regions	Zhetysu	3	0 (0.0)	0.0–56.1	0 (0.0)	0.0–56.1
			Almaty	2	0 (0.0)	0.0–65.7	0 (0.0)	0.0–65.7
			Zhambyl	10	0 (0.0)	0.0–27.7	0 (0.0)	0.0–27.7
			Turkestan	9	1 (11.0)	1.9–43.5	1 (11.0)	1.9–43.5
	2024, Spring		Zhetysu	3	0 (0.0)	0.0–56.1	3 (100.0)	43.9–100
			Almaty	15	0 (0.0)	0.0–20.3	6 (40.0)	19.8–64.2
			Zhambyl	16	14 (87.5)	63.9–96.5	15 (93.8)	71.7–98.9
			Turkestan	16	4 (25.0)	10.1–49.5	7 (43.8)	23.1–66.8
	2023, Autumn	Northern regions	Qostanay	2	0 (0.0)	0.0–65.7	0 (0.0)	0.0–65.7
	2024, Spring		Pavlodar	6	0 (0.0)	0.0–39.0	0 (0.0)	0.0–39.0
			North Kazakhstan	5	0 (0.0)	0.0–43.4	0 (0.0)	0.0–43.4
Cattle	2023, Autumn	Southern regions	Zhetysu	0	0 (0.0)	0	0 (0.0)	0
			Almaty	16	1 (6.3)	1.1–28.3	1 (6.3)	1.1–28.3
			Zhambyl	33	2 (6.0)	1.7–19.6	1 (3.0)	0.5–15.3
			Turkestan	27	5 (18.5)	8.2–36.7	5 (18.5)	8.2–36.7
	2024, Spring		Zhetysu	6	1 (16.7)	3.0–56.3	4 (66.7)	30.0–90.3
			Almaty	18	10 (55.5)	33.7–75.4	8 (44.4)	24.6–66.3
			Zhambyl	56	11 (19.6)	11.3–31.8	15 (26.8)	17.0–39.6
			Turkestan	16	0 (0.0)	0.0–19.4	6 (37.5)	18.5–61.4
	2023, Autumn	Northern regions	Qostanay	21	0 (0.0)	0.0–15.5	0 (0.0)	0.0–15.5
	2024, Spring		Pavlodar	25	5 (20.0)	8.9–39.1	0 (0.0)	0.0–13.3
			North Kazakhstan	25	0 (0.0)	0.0–13.3	0 (0.0)	0.0–13.3

In northern regions, overall seroprevalence was zero in samples collected in Autumn 2023. In 2024, the overall seroprevalence was 0.6% (95% CI: 0.1–3.2) for sheep and 10.0% (95% CI: 4.3–21.4) for cattle, with no seroprevalence in goats (Supplementary Table 4). Detailed serological prevalence by oblasts is presented in Table 1.

rRT-PCR testing revealed that the number of positive samples for bluetongue virus (BTV) RNA was significantly higher in southern regions compared to northern regions (*p* < 0.0001). Additionally, the overall number of rRT-PCR-positive samples was higher in 2024 than in 2023 (Supplementary Table 2). BTV RNA was detected in the different animal species as follows: sheep-−8.9% (95% CI: 6.9–11.3; *p* = 0.57), goats-−36.8% (95% CI: 27.4–47.3), and cattle-−16.5% (95% CI: 12.3–21.6). Notably, the number of rRT-PCR-positive samples exceeded those identified as positive by ELISA (Supplementary Table 3).

In the southern regions in 2023, the proportions of rRT-PCR-positive results were: sheep-−7.2% (95% CI: 4.2–12.1), goats-−4.2% (95% CI: 0.7–20.2), and cattle-−9.2% (95% CI: 4.5–17.8). In 2024, the percentage of rRT-PCR-positive samples among sheep increased to 17.0% (95% CI: 12.9–22.0), among goats to 62.0% (95% CI: 48.1–74.1), and among cattle to 34.4% (95% CI: 25.6–44.3; Supplementary Table 4). Conversely, in the northern regions, no rRT-PCR-positive animals for BTV were detected in either 2023 or 2024 (Supplementary Table 4). The distribution of BTV RNA detection across oblasts is presented in Table 1.

### Distribution of *Culicoides* spp. in the southern and northern regions of Kazakhstan

**Figure 2:**
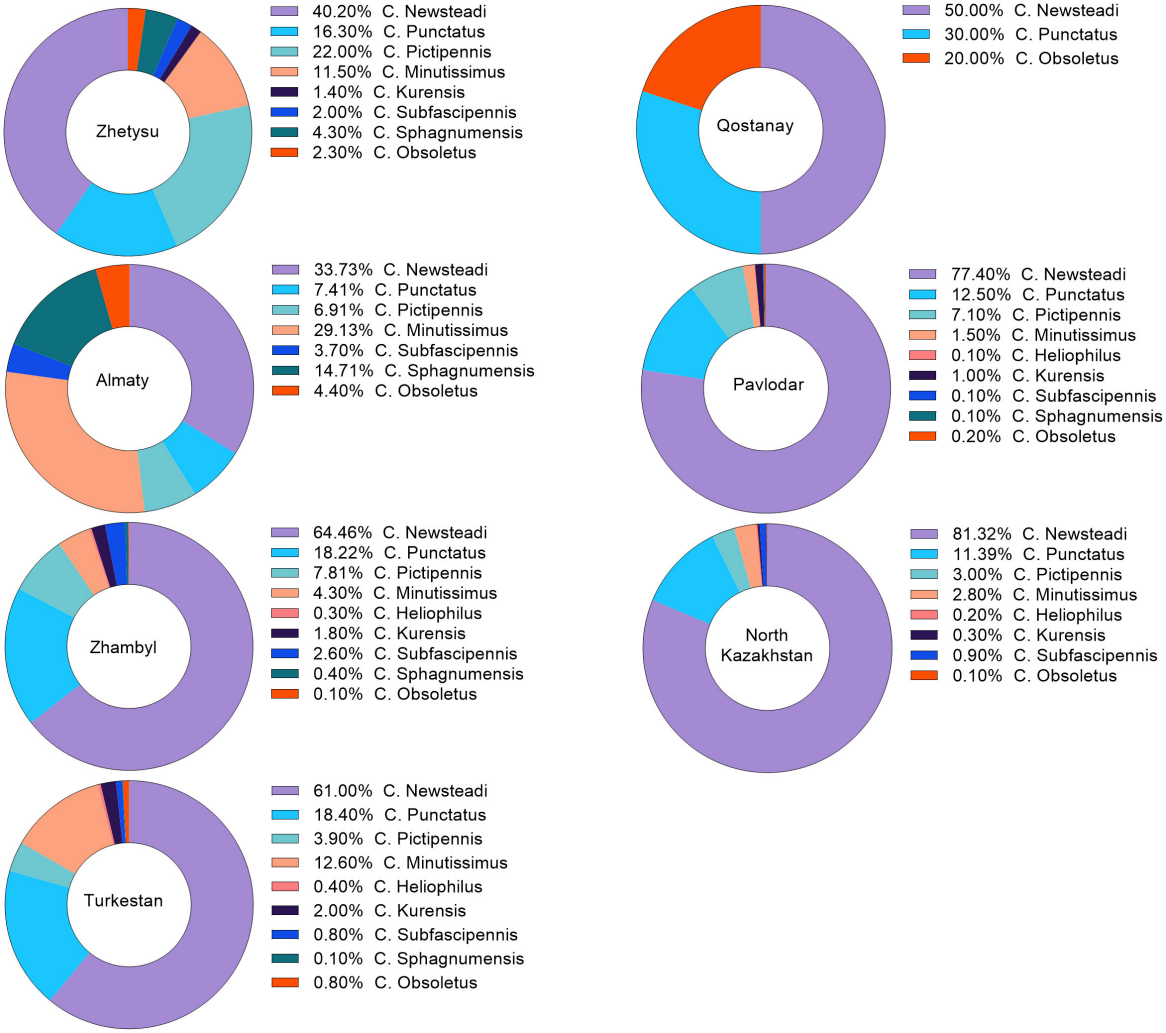
Percentage distribution of *Culicoides* species occurrence by region. The percentage distribution of *Culicoides* species occurrence was calculated based on the captured species. This percentage may vary according to the differing weather conditions in each region.

A total of 11,859 *Culicoides* spp. were collected in the southern and northern regions of Kazakhstan. Specifically, 209 specimens (1.8%) were from the Zhetysu, 564 (4.8%) from the Almaty oblast, 2,201 (18.6%) from the Turkestan, 6,350 (53.5%) from the Zhambyl, 1,287 (10.9%) from the North Kazakhstan oblast, and 1,248 (10.5%) from the Pavlodar oblast. Detailed quantitative and percentage metrics of captured *Culicoides* in each oblast are presented in Figure 2 and Table 2. The observed variations in the capture rates of *Culicoides* across different oblasts are attributed to changes in weather conditions.

**Table 2:** Quantitative metrics of *Culicoides* capture in the southern and northern regions of Kazakhstan for 2024.

**Oblast (Administrative division)**	**Rural district, Village (Administrative division)**	**Species and number of midges, pcs**									**Total number**
		**Newsteadi**	**Punctatus**	**Pictipennis**	**Minutissimus**	**Heliophilus**	**Kurensis**	**Subfascipennis**	**Sphagnumensis**	**Obsoletus**	
Zhetysy	Koksu	13	11	19	9	–	1	2	4	3	62
	Kerbulaq	71	23	27	15	–	2	2	5	2	147
**Total**		84	34	46	24	–	3	4	9	5	209 (1.8%)
Almaty	Tasqarasu, Tasqarasu village	78	24	18	92	–	–	12	11	8	243
	Mezhdurechensky, Aqsy village	112	18	21	72	–	–	9	72	17	321
**Total**		190	42	39	164			21	83	25	564 (4.8%)
Turkestan	Sholaqkorgan, Sholaqkorgan village	476	208	15	117	4	23	11	2	4	860
	Saryagash, Saryagash city	552	162	42	98	2	14	2	1	11	884
	Qyzylzhar, Zhaskeldi village	315	35	28	63	3	7	4	–	2	457
**Total**		1,343	405	85	278	9	44	17	3	17	2,201
Zhambyl	Qarakemer, Qarakemer village	415	15	108	24	5	38	118	4	1	728 (18.6%)
	Qarasu, Ashybulaq village	852	42	46	52	2	11	21	2	1	1,029
	Saudakent, Baiqadam village	542	172	43	18	–	2	10	12	3	802
	Zhaiylma, Zhaiylma village	792	381	82	92	7	44	13	4	–	1,415
	Merke, Merke village	688	212	35	21	3	11	2	1	2	975
	Tolebi, Tolebi village	746	317	169	46	–	7	4	1	–	1,290
	Zhanaqogam, Koktobe village	13	–	–	–	–	–	–	–	–	13
	Asa, Asa village	43	18	11	22	–	2	–	–	2	98
**Total**		4,091	1,157	579	275	17	115	168	24	9	6,350 (53.5%)
North Kazakhstan	Avangard, Roshchino village	245	62	–	–	–	2	–	–	1	310
	Bulaevo city	372	43	18	8	2	2	–	–	–	445
	Talshyk, Talshyk village	112	–	12	21	–	–	8	–	–	153
	Telzhan, Telzhan village	318	42	9	7	–	–	3	–	–	379
**Total**		1,047	147	39	36	–	4	11		1	1,287 (10.9%)
Pavlodar	Kentubek, Kentubek village	215	71	57	–	–	3	1	–	2	349
	Koktobe, Koktobe village	304	–	32	12	1	7	–	1	–	357
	Toraygyr, Toraygyr village	182	53	–	–	–	3	–	–	–	238
	Aksan, Aksan village	265	32	–	7	–	–	–	–	–	304
**Total**		966	156	89	19	1	13	1	1	2	1,248 (10.5%)
**Total**		7,531	1,941	877	796	29	179	222	120	59	11,859

### Morphological and molecular identification of *Culicoides* spp.

**Figure 3:**
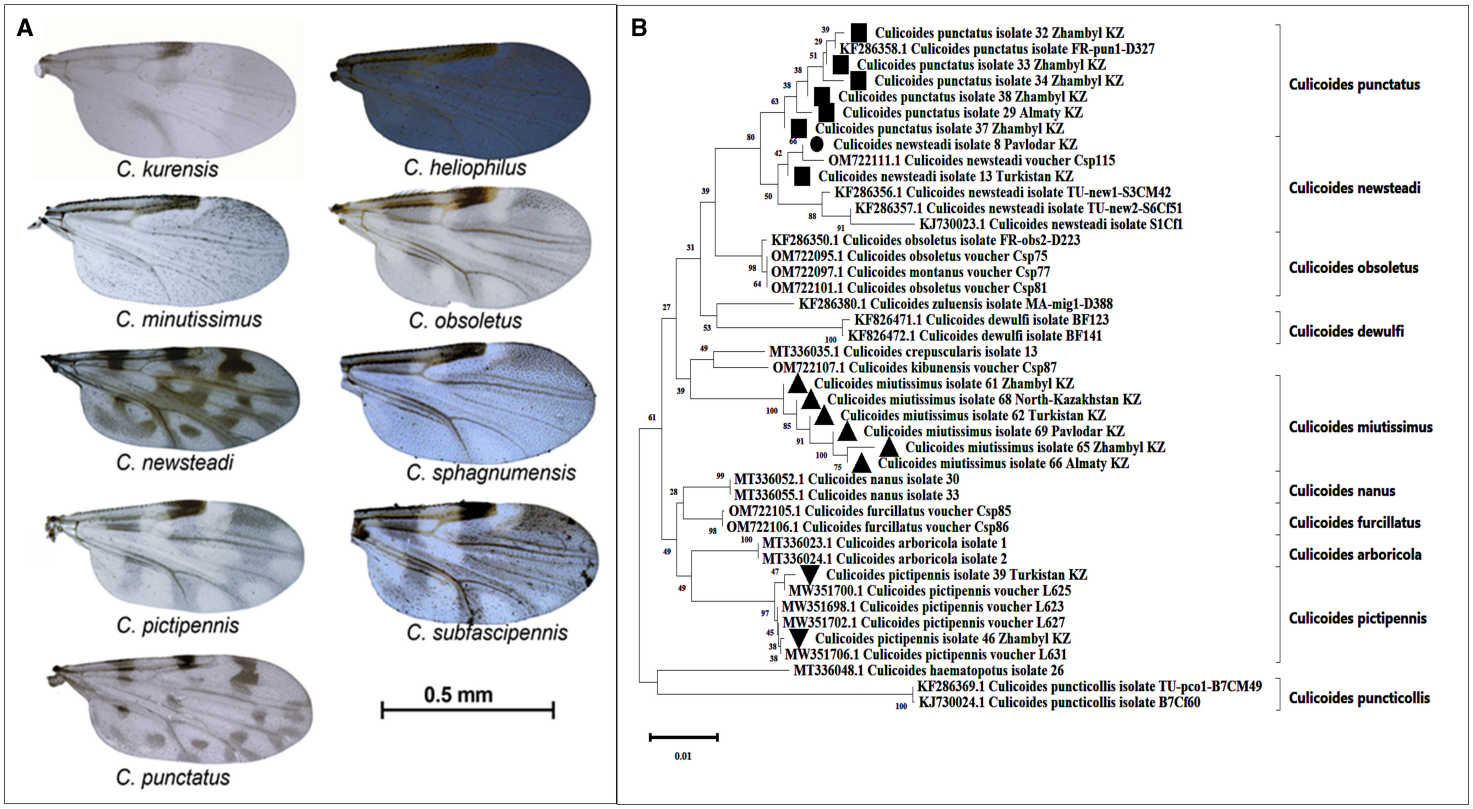
Morphological and Genetic Identification of *Culicoides* spp. **(A)**—Wings of *Culicoides* species captured in Kazakhstan. Photographs of the wings were taken using a camera mounted on a stereomicroscope (Ken-A-Vesion, USA) and processed with specialized software, PowerPoint. Scale bar: 0.5 mm. **(B)**—Phylogenetic tree of *Culicoides* species captured in the southern and northern regions of Kazakhstan. Evolutionary relationships of the taxa were analyzed using the Neighbor-Joining method (30). The optimal phylogenetic tree displays the relationships among 43 nucleotide sequences. The reliability of taxa clustering was assessed using a bootstrap test (1,000 iterations), with corresponding percentages indicated next to the branches (31). The branch lengths on the tree are proportional to evolutionary distances, which were calculated using the Tamura-Nei method (32) and expressed as the number of nucleotide substitutions per site. Compositional bias differences among sequences were taken into account in evolutionary comparisons (32).

According to the morphological identification based on wing patterns, the following nine species of *Culicoides* were identified (Figure 3A): *C. Newsteadi, C. Punctatus, C. Pictipennis, C. Minutissimus, C. Heliophilus, C. Kurensis, C. Subfascipennis, C. Sphagnumensis, C. Obsoletus*. Among them, the most dominant species were *C. Newsteadi, C. Punctatus, C. Pictipennis, C. Minutissimus* (Figure 2).

During the molecular identification, it was confirmed that the species *C. newsteadi, C. punctatus, C. pictipennis*, and *C. minutissimus* aligned with their morphological identification (Figure 3B). Five other species (*C. heliophilus, C. kurensis, C. subfascipennis, C. sphagnumensis, and C. obsoletus*) could not be confirmed molecularly due to insufficient quality and or quantity of data obtained.

### Prevalence of BTV in *Culicoides* species

Morphological and genetic identification of *Culicoides* species facilitated the creation of 79 pooled samples categorized by species and collection sites. The number of pooled samples collected in each region is presented in Table 3.

**Table 3:** Detection of BTV RNA in *Culicoides* species.

**Oblast**	**Species**	**Number of examined pools^#^**	**Number of rRT-PCR-positive pools**	Ct value range^*^	Potential host-feeders^**^
North Kazakhstan	*C. Newsteadi*	4	0	38.0–39.4	Cattle, Sheep
	*C. Punctatus*	3	0	39.4–41.0	Goat, Cattle
	*C. Pectipennis*	3	0	38.9–39.7	Goat
	*C. Miutissimus*	3	0	39.7–40.1	Cattle
Pavlodar	*C. Newsteadi*	4	0	39.7–41.5	Sheep, Goat, Cattle
	*C. Punctatus*	3	0	37.1–43.2	
	*C. Pectipennis*	2	0	36.9–41.1	Cattle, Sheep
	*C. Miutissimus*	2	0	39.1–39.3	
Almaty	*C. Newsteadi*	2	0	37.8–39.4	Cattle, Sheep
	*C. Punctatus*	2	0	39.1–39.5	
	*C. Pectipennis*	1	0	39.7–41.5	
	*C. Miutissimus*	2	2	34.5–34.6	Sheep
	*C. Sphagnumennis*	2	1	33.4	
Turkestan	*C. Newsteadi*	3	0	37.8–39.4	Goat, Sheep
	*C. Punctatus*	3	0	39.1–39.5	Sheep, Goat, Cattle
	*C. Pectipennis*	3	0	37.8–39.4	
	*C. Miutissimus*	3	2	31.1–33.5	
	*C. Kurensis*	2	0	38.9–42.4	
Zhambyl	*C. Newstedi*	7	2	33.1–35.6	Goat, Sheep
	*C. Punctatus*	7	0	37.8–40.1	Cattle, Sheep
	*C. Pectipennis*	7	3	31.2–35.1	Cattle, Sheep
	*C. Miutissimus*	6	4	32.3–35.6	Sheep, Goat, Cattle
	*C. Sphagnumennis*	3	2	35.4–35.6	Goat, Sheep
	*C. Kurensis*	2	0	39.0–41.1	Sheep
**Total**	79	16	–	–

(#) Each pool contained between 5 and 50 midges, depending on the total number of captured specimens. (^*^)—Values up to 35.9 were considered positive, values from 36.0 to 39.9 were considered suspicious, and values of 40.0 and above were considered negative. (^**^)—The midges were captured from the premises where these animals were housed.

During the study, no BTV RNA was detected in *Culicoides* specimens collected from the North Kazakhstan and Pavlodar regions. In the Almaty region, RNA was identified in *C. miutissimus* (Ct 34.5–34.6) and *C. sphagnumennis* (Ct 33.4). In contrast, in the Turkestan region, RNA was detected only in *C. miutissimus* (Ct 31.1–33.5).

In the Zhambyl region, BTV RNA was identified in the following midge species: *C. newsteadi* (Ct 33.1–35.6), *C. pectipennis* (Ct 31.2–35.1), *C. miutissimus* (Ct 32.3–35.6), and *C. sphagnumennis* (Ct 35.4–35.6; see Table 3).

## Discussion

This study provides critical data on the latest seroprevalence of BT and identification of potential BTV vectors in Kazakhstan. In this study, 972 blood and serum samples were collected from sheep, goat and cattle from southern and northern Kazakhstan in Autumn 2023 and Spring 2024. The testing results indicated that seroprevalence and virus distribution were higher in the southern regions compared to the northern regions. It is important to note that sample collection was based on an estimated seroprevalence of ~5%. According to previous studies (15–18), the seroprevalence in the southern regions of Kazakhstan was at least 20%. However, the results of this study confirmed the proposed hypothesis, demonstrating a seroprevalence rate of 5% or higher. The overall seroprevalence of BTV across all animal species was as follows: 4.2% among sheep, 21.8% among goats, and 14.4% among cattle. It is important to note that, despite the trend of higher seroprevalence in goats and cattle, no statistically significant differences were observed among the animal species (*p* > 0.05). Compared to previous years, the seroprevalence in the southern regions increased across all animal species in 2024 compared to 2023. This increase was particularly notable among goats (from 3.8% in 2023 to 29.5% in 2024) and cattle (from 7.8% to 18.5%). In the northern regions, seroprevalence was zero for all animal species in 2023. However, in 2024, seroprevalence of 10.0% was observed in cattle, indicating the onset of virus circulation in these regions.

To confirm the serological data obtained, rRT-PCR testing was conducted. The rRT-PCR results corroborated the findings from ELISA, demonstrating a significantly higher number of positive BTV RNA samples in the southern regions compared to the northern regions (*p* < 0.0001), highlighting active virus circulation in these areas. It is noteworthy that the number of rRT-PCR-positive samples was considerably higher in 2024 than in 2023. For instance, the proportion of rRT-PCR-positive samples in sheep increased from 7.2% in 2023 to 17.0% in 2024, while in cattle, the increase was from 9.2% to 34.4%. A particularly sharp rise in rRT-PCR-positive samples was observed among goats, where the percentage rose from 4.2% in 2023 to 62.0% in 2024. This growth may be associated with more intense virus circulation among this species or changes in risk factors. Additionally, in some cases, the number of PCR-positive samples exceeded the number of ELISA seropositive samples. This could indicate recent infections, where animals had not yet developed antibodies, or reflect the higher sensitivity of rRT-PCR in detecting active infections.

In this study, sequencing and virus isolation from BTV rRT-PCR-positive samples were not performed due to financial and resource limitations. However, given the importance, detailed characterization of circulating strains will be focused in future studies. The information on the genetic diversity of BTV in Kazakhstan would help to trace the origin and spread of BTV within different ecological and climatic zones within the country.

The presence and the abundance of *Culicoides* midges, influence iBTV transmission, and understanding their regional diversity is essential for assessing the risk of BTV spread and designing effective control measures.

An ~1,400 species of *Culicoides* midges have been identified globally, with 130 species reported in the former USSR. Among these, *C. obsoletus, C. dewulfi, C. scoticus, C. pulicaris, C. stigma, C. nubeculosus, C. chiopterus*, and *C. punctatus* are widely distributed in Russia, with their range extending up to 72° latitude (26). In Kazakhstan, members of *Culicoides* subgenera *Avaritia, Culicoides, Hofmania, Monoculicoides, Oaecata, Wirthomyia, Silvaticulicoides* (*Beltranmyia* have been reported (26, 33)). According to the literature, the most abundant species in southern Kazakhstan are *C. punctatus, C. newsteadi*, and *C. obsoletus* (34, 35). In central Kazakhstan, *C. obsoletus, C. dewulfi*, and *C. montanus* dominate (36); in northern Kazakhstan, *C. obsoletus, C. punctatus*, and *C. chiopterus* are prevalent (33, 36); while in eastern Kazakhstan, *C. grisescens* and *C. obsoletus* are the most common (33, 36).

According to the literature, *C. imicola* is the primary vector of BTV in Africa, the Middle East, and southern Europe, *C. sonorensis* in North America, and *C. brevitarsis* in Australia. It is worth noting that *C. imicola* is absent from the listed species in Kazakhstan; however, *C. obsoletus* a known BTV vector in European countries, is present in Kazakhstan. In Europe, the main BTV vectors are midges from the *obsoletus* and *pulicaris* complexes (6). *C. pulicaris* was not detected in any region during this study.

Adult females *Culicoides* require blood during their gonotrophic cycle. Therefore, they feed on warm-blooded animals every 3–4 days, staying close to the ground. Different species exhibit distinct host preferences: some are anthropophilic, feeding on humans, others are zoophilic, preferring livestock, or ornithophilic, feeding on birds. Their activity patterns also vary, with some being active predominantly at twilight and nighttime, while others feed during the day, either in open spaces or livestock enclosures. Based on the samples collected in this study, host preferences of the *Culicoides* were; Sheep-−42%, Cattle-−34%, and Goats-−24%.

In this study, *nine Culicoides* spp. were morphologically identified and the identity of four of the species were confirmed by sequencing 28S rDNA. The 28S marker, is commonly employed in examining the evolutionary relationships among insects. Nevertheless, the effectiveness of the primer employed for amplifying this marker was observed to be diminished in relation to some species. This issue may be attributable to the genetic traits of the taxa influencing the primer binding process, the presence of polymorphisms in the amplification regions specific to these species, or the degradation or low quality of the DNA in the initial samples.

For further research, it is planned to revise the approach to primer selection, including the use of alternative sequences that are more specific to the species in question, or to modify the PCR conditions to improve amplification efficiency.

Out of the nine *Culicoides* species four (*C. Miutissimus, C. Newstedi, C. Pectipennis and C. Sphagnumennis*) were identified as potential BTV vectors in Kazakhstan. In southern regions of Kazakhstan, BTV RNA was primarily detected in *C. miutissimus* and *C. sphagnumennis*. These findings align with previous studies indicating active virus circulation in these warm and humid regions (34, 35). These species play a critical role in the epidemiological situation in the southern regions, as the environmental conditions there are favorable for their reproduction and activity (33).

In northern Kazakhstan, specifically in regions such as North Kazakhstan and Pavlodar, viral RNA was not detected in any *Culicoides* spp., likely due to less favorable climatic conditions for the vectors in these areas (18). Climatic factors, such as cold winters and relatively dry summer months, limit their activity and ability to transmit the virus.

As for *C. newstedi* and *C. pectipennis*, they exhibited a high level of BTV infection in the Zhambyl region, strongly suggesting their potential role as vectors (34). Previously, these species have also been reported in Russia and Central Asia as important BTV carriers (1).

Additionally, the literature indicates that *C. imicola* and *C. obsoletus*, though not widely present in Kazakhstan, are primary vectors of BTV in Europe and Africa (6). These species possess significant epidemiological potential in the global spread of the virus, and if they were to be detected in Kazakhstan, their role in the epidemiological situation could become crucial.

Notably, despite the detection of seropositive animals for BT in the northern regions of Kazakhstan, no PCR-positive animals or vectors were found among susceptible livestock (sheep, goats, and cattle). The presence of seropositive animals in could possibly be explained through animal movement of seropositive animals from the southern regions to the northern regions, or lower level of BT infections. In contrast, the BT situation in southern regions was the presence of both seropositive and PCR-positive animals, as well as PCR-positive midges, were identified. In all southern regions, the RNA of BTV was predominantly detected in *C. miutissimus* among PCR-positive *Culicoides* species. However, the prevalence of *C. miutissimus* in the southern regions varied significantly, ranging from 4.3% to 29.1% (see Figure 2).

These findings have significant and multifaceted implications for the epidemiology of the diseases they transmit (37). Analyzing the obtained results, we aimed to assess the current epidemiological situation of BT in the northern and southern regions of Kazakhstan. However, this study has highlighted the need for a more comprehensive analysis taking into account the climatic heterogeneity of the country. For a more accurate assessment of the risk of virus transmission, it is necessary to consider multiple factors, including climate zones, temperature, the presence and density of susceptible animals, age, sex, vector populations, insect biological activity, prevailing wind directions, and other epidemiologically significant parameters (18, 33, 38).

According to previous mathematical modeling studies (18), it has been scientifically established that the spread of Bluetongue virus is most likely in the southern and southeastern regions of Kazakhstan. The results obtained in our study confirm this prediction, demonstrating a high level of seroprevalence in these areas. This pattern may be associated with favorable climatic conditions that facilitate virus circulation and vector activity. A study conducted in Southern Italy (Campania region) demonstrated that moderately mild winters and high seasonal *Culicoides* activity contribute to sustained virus circulation (39). Similarly, a meta-analysis of BTV seroprevalence in Africa (40) emphasized that the year-round warm climate and high *Culicoides* population density ensure continuous virus transmission among various animal species, including cattle, sheep, goats, and camels. However, unlike these regions, BTV circulation in Kazakhstan is restricted exclusively to the warm season, necessitating the development of region-specific control and prevention measures.

Furthermore, the transboundary spread of BTV remains a significant risk factor. However, data on the epidemiological situation in neighboring countries bordering our study regions, such as Russia, Kyrgyzstan, and Uzbekistan, are extremely limited. Therefore, future research should incorporate an assessment of the epizootic situation in border areas and the influence of climatic factors on virus circulation.

Nevertheless, the data obtained not only confirm the current distribution of vectors but also allow for the prediction of BTV dynamics in the northern and southern regions of Kazakhstan. This, in turn, may contribute to the improvement of preventive and anti-epizootic measures in the future.

## Conclusion

The results of this study highlight the importance of systematic monitoring of *Culicoides* spp. in Kazakhstan. The identification of species such as *C. miutissimus, C. sphagnumennis, C. newstedi*, and *C. pectipennis* as potential BTV vectors calls for further investigation to develop effective measures to prevent the spread of BTV in the region. The abundance of *Culicoides* species can vary during the vector season. This study was conducted in the Spring and the Autumn. Therefore, in future studies samples collected in summer months might provide information on additional species of *Culicoides* that could serve as BTV vectors. To confirm the vector competence of *Culicoides* species, it is essential to demonstrate not only their ability to acquire the virus from a host but also their capacity to retain and transmit it to a new, uninfected susceptible host. Therefore, detecting the virus in the body of a blood-feeding insect alone is insufficient evidence of its vector competence. Hence, a more precise evaluation requires separate analysis of the insect's head, salivary glands, and body. Additionally, it is crucial to consider climatic and ecological factors that influence vector activity and their ability to transmit the virus. These factors must be integrated into future studies to provide a comprehensive understanding of vector behavior and disease transmission dynamics.

## Data Availability

The original contributions presented in the study are included in the article/Supplementary material, further inquiries can be directed to the corresponding author/s.
