# Supplementary material for: Prevalence of Bluetongue and the distribution of Culicoides species in northern and southern regions of Kazakhstan in 2023–2024
Source: Front Vet Sci. 2025 Mar 6;12:1559636. doi: 10.3389/fvets.2025.1559636 (PMC11924940; doi:10.3389/fvets.2025.1559636)
Supplement: Supplementary file 1 [file Table_1.docx]

Table S1 – Number (Sample Size) of Administrative and Epizootiological Units and Animals to be Examined Depending on Prevalence and a Confidence Level of 0.95

| Number of Animals, Heads |  | Prevalence of Animals, % | | | | | | | |
| --- | --- | --- | --- | --- | --- | --- | --- | --- | --- |
|  |  | 1 | 2 | 5 | 10 |  | 20 | 25 | 30 |
| Less than 10 |  | If the number of animals is less than 10, samples are taken from all animals. | | | |  | Calculated by Formula | | |
| 10 |  | 10 | 10 | 10 | 10 |  | 8 | 7 | 7 |
| 20 |  | 20 | 20 | 20 | 16 |  | 11 | 9 | 8 |
| 30 |  | 30 | 30 | 26 | 19 |  | 11 | 9 | 8 |
| 40 |  | 40 | 40 | 33 | 22 |  | 13 | 10 | 9 |
| 50 |  | 50 | 50 | 37 | 23 |  | 13 | 10 | 9 |
| 60 |  | 60 | 58 | 40 | 24 |  | 13 | 11 | 9 |
| 70 |  | 70 | 65 | 42 | 25 |  | 13 | 11 | 9 |
| 80 |  | 80 | 72 | 44 | 26 |  | 14 | 11 | 9 |
| 90 |  | 90 | 77 | 46 | 26 |  | 14 | 11 | 9 |
| 100 |  | 100 | 82 | 47 | 27 |  | 14 | 11 | 9 |
| 120 |  | 116 | 90 | 49 | 27 |  | 14 | 11 | 9 |
| 140 |  | 130 | 97 | 51 | 28 |  | 14 | 11 | 9 |
| 160 |  | 143 | 102 | 52 | 28 |  | 14 | 11 | 9 |
| 180 |  | 154 | 107 | 53 | 28 |  | 14 | 11 | 9 |
| 200 |  | 164 | 111 | 54 | 28 |  | 14 | 11 | 9 |
| 250 |  | 184 | 118 | 55 | 29 |  | 14 | 11 | 9 |
| 300 |  | 199 | 124 | 56 | 29 |  | 14 | 11 | 9 |
| 350 |  | 212 | 128 | 57 | 29 |  | 14 | 11 | 9 |
| 400 |  | 222 | 131 | 58 | 29 |  | 15 | 11 | 9 |
| 450 |  | 230 | 133 | 58 | 30 |  | 15 | 11 | 9 |
| 500 |  | 237 | 136 | 59 | 30 |  | 15 | 11 | 9 |
| 600 |  | 248 | 139 | 59 | 30 |  | 15 | 12 | 9 |
| 700 |  | 256 | 141 | 60 | 30 |  | 15 | 12 | 9 |
| 800 |  | 262 | 143 | 60 | 30 |  | 15 | 12 | 9 |
| 900 |  | 268 | 144 | 60 | 30 |  | 15 | 12 | 9 |
| 1000 |  | 272 | 146 | 60 | 30 |  | 15 | 12 | 9 |
| 1200 |  | 278 | 147 | 61 | 30 |  | 15 | 12 | 9 |
| 1400 |  | 283 | 149 | 61 | 30 |  | 15 | 12 | 9 |
| 1600 |  | 287 | 150 | 61 | 30 |  | 15 | 12 | 9 |
| 1800 |  | 290 | 150 | 61 | 30 |  | 15 | 12 | 9 |
| 2000 |  | 292 | 151 | 61 | 30 |  | 15 | 12 | 9 |
| 3000 |  | 299 | 153 | 61 | 30 |  | 15 | 12 | 10 |
| 4000 |  | 303 | 154 | 62 | 30 |  | 15 | 12 | 10 |
| 5000 |  | 305 | 154 | 62 | 30 |  | 15 | 12 | 10 |
| 6000 |  | 307 | 155 | 62 | 30 |  | 15 | 12 | 10 |
