# Supplementary material for: Prevalence of Bluetongue and the distribution of Culicoides species in northern and southern regions of Kazakhstan in 2023–2024
Source: Front Vet Sci. 2025 Mar 6;12:1559636. doi: 10.3389/fvets.2025.1559636 (PMC11924940; doi:10.3389/fvets.2025.1559636)
Supplement: Supplementary file 2 [file Table_2.docx]

Table S2 – Prevalence of Bluetongue Virus by Regions of Kazakhstan

| Region | Year | Number of animals  (herd) | Seropositive Samples (n, %) | 95 % Confidence Intervals (CI) | rRT-PCR-Positive Samples (n, %) | 95 % Confidence Intervals (CI) |
| --- | --- | --- | --- | --- | --- | --- |
| Southern Regions | 2023 | 267 | 18 (6,7) | 4.3-10.4 | 20 (7,5) | 4.9-11.3 |
|  | 2024 | 411 | 58 (14,1) | 11.1-17.8 | 109 (26,5) | 22.5-31.0 |
| Northern Regions | 2023 | 62 | 0 (0,0) | 0.0 | 0 (0,0) | 0 |
|  | 2024 | 232 | 6 (2,6) | 1.2-5.5 | 0 (0,0) | 0 |
