# Supplementary material for: Prevalence of Bluetongue and the distribution of Culicoides species in northern and southern regions of Kazakhstan in 2023–2024
Source: Front Vet Sci. 2025 Mar 6;12:1559636. doi: 10.3389/fvets.2025.1559636 (PMC11924940; doi:10.3389/fvets.2025.1559636)
Supplement: Supplementary file 3 [file Table_3.docx]

Table S3 – Overall Prevalence of Bluetongue Virus by Animal Species in Kazakhstan for 2023–2024

| Animal species | Number of animals  (herd) | Seropositive Samples (n, %) | 95 % Confidence Intervals (CI) | rRT-PCR-Positive Samples (n, %) | 95 % Confidence Intervals (CI) |
| --- | --- | --- | --- | --- | --- |
| Sheep | 642 | 27 (4.2) | 2.9-6.0 | 57 (8.9) | 6.9-11.3 |
| Goat | 87 | 19 (21.8) | 14.4-31.6 | 32 (36.8) | 27.4-47.3 |
| Cattle | 243 | 35 (14.4) | 10-5-19.4 | 40 (16.5) | 12.3-21.6 |
