# Supplementary material for: Prevalence of Bluetongue and the distribution of Culicoides species in northern and southern regions of Kazakhstan in 2023–2024
Source: Front Vet Sci. 2025 Mar 6;12:1559636. doi: 10.3389/fvets.2025.1559636 (PMC11924940; doi:10.3389/fvets.2025.1559636)
Supplement: Supplementary file 4 [file Table_4.docx]

Table S4 – Prevalence of Bluetongue Virus by Year Among Different Animal Species in the Southern and Northern Regions of Kazakhstan

| Year | Region | Animal species | Number of animals  (herd) | Seropositive Samples (n, %) | 95 % Confidence Intervals (CI) | rRT-PCR-Positive Samples (n, %) | 95 % Confidence Intervals (CI) |
| --- | --- | --- | --- | --- | --- | --- | --- |
| 2023 | Southern Regions | Sheep | 167 | 9 (5,4) | 2,9-9,9 | 12 (7,2) | 4,2-12,1 |
|  |  | Goats | 24 | 1 (4,2) | 0,7-20,2 | 1 (4,2) | 0,7-20,2 |
|  |  | Cattle | 76 | 8 (10,5) | 5,4-19,4 | 7 (9,2) | 4,5-17,8 |
|  | Northern Regions | Sheep | 39 | 0 (0,0) | 0 | 0 (0,0) | 0 |
|  |  | Goats | 2 | 0 (0,0) | 0 | 0 (0,0) | 0 |
|  |  | Cattle | 21 | 0 (0,0) | 0 | 0 (0,0) | 0 |
| 2024 | Southern Regions | Sheep | 265 | 18 (6,8) | 4,3-10,5 | 45 (17,0) | 12,9-22,0 |
|  |  | Goats | 50 | 18 (36,0) | 24,1-49,9 | 31 (62,0) | 48,1-74,1 |
|  |  | Cattle | 96 | 22 (22,9) | 15,6-32,3 | 33 (34,4) | 25,6-44,3 |
|  | Northern Regions | Sheep | 171 | 1 (0,6) | 0,1-3,2 | 0 (0,0) | 0 |
|  |  | Goats | 11 | 0 (0,0) | 0 | 0 (0,0) | 0 |
|  |  | Cattle | 50 | 5 (10,0) | 4,3-21,4 | 0 (0,0) | 0 |
